# Resistance to single dose albendazole and reinfection with intestinal helminths among children ages 2 to 11 years from the Peruvian Amazon region: a study protocol

**DOI:** 10.1186/s12879-022-07494-0

**Published:** 2022-06-07

**Authors:** Greisi Curico, Paul García-Bardales, Tackeshy Pinedo, Wagner Shapiama, Miguel Moncada-Yaicate, Lucero Romaina, Pablo P. Yori, Maribel Paredes-Olortegui, Graciela Meza-Sánchez, Andrés G. Lescano, Valerie A. Paz-Soldan, Francesca Schiaffino, Richard A. Oberhelman, Margaret N. Kosek

**Affiliations:** 1 Laboratorio Satelite Iquitos, Asociación Benéfica Prisma, Área de Investigaciones Biomédicas, Calle Ramirez Hurtado Nº 622, Iquitos, Peru; 2 Division of Infectious Diseases and International Health, Department of Internal Medicine, University of Virginia, MR-6 Rm 2207, 345 Crispell Dr, Charlottesville, VA 22908 USA; 3 Universidad Nacional de la Amazonia Peruana, Jirón Nauta, 16002 Iquitos, Peru; 4 Facultad de Salud Pública y Administración, Universidad Peruana Cayetano Heredia, Av. Honorio Delgado, 430. San Martin de Porres, Lima, Peru; 5 Department of Tropical Medicine, Tulane School of Public Health and Tropical Medicine, 1440 Canal Street, Suite 2310, New Orleans, LA 70112 USA

**Keywords:** Helminths, Deworming, Albendazole, Ivermectin, Children, Peru

## Abstract

**Background:**

Deworming programs aimed at reducing morbidity and mortality from geohelminth infections are common in many countries where these infections are endemic, but data demonstrating increasing levels of resistance to albendazole and mebendazole are causes for concern. Studies to evaluate the clinical efficacy of deworming programs are critical to maintain high infection control goals.

**Methods:**

We propose to assess the clinical efficacy of Peruvian national guidelines for deworming programs in a prospective observational study conducted in the Amazon River basin area near Iquitos, Peru. Major outcomes to be evaluated include (1) albendazole resistance of intestinal helminths (trichuriasis, ascariasis, hookworm), and (2) frequency of reinfection with intestinal helminths 4 months after treatment with albendazole. Children ages 2–11 years from the Belén District of Iquitos will be identified based on a community census. Following parental informed consent, demographic data, weight, and height will be recorded and a stool specimen for parasitological exam by direct observation and Kato-Katz concentration method, and helminthic egg counts will be collected prior to administration of albendazole, following Peruvian national guidelines. Follow-up stool specimens examined in the same manner will be collected at 20 days, 90 days, and 100 days following initial administration of albendazole, and based on parasites found repeat treatment will be administered in accordance with national guidelines. Real-time multiplex qPCR will be performed on helminth positive samples collected prior to initial deworming and on helminth-positive specimens detected on day 15–20. A total sample size of 380 participants was calculated based on total population in the target group and prevalence estimates of helminth infections and clinical resistance based on recent data.

**Discussion:**

Data from observational clinical efficacy studies are important to guide geohelminth infection control programs.

*Trial registration* https://www.researchregistry.com/. Identification number: researchregistry7736; Registered retrospectively March 13, 2022; https://www.researchregistry.com/browse-the-registry#home/registrationdetails/622e024cf06132001e3327bf/

## Background

Worldwide, approximately 1.5 billion people or about 24% of the world’s population, are infected with soil-transmitted helminths (STH) [1]. Soil-transmitted helminth infections are widely distributed in tropical and subtropical areas, especially in sub-Saharan Africa, America, China and East Asia. More than 267 million preschool-age children and more than 568 million school-age children live in areas with intense transmission and are able to benefit from treatment and prevention strategies [2]. In low- and middle-income countries, risk factors for STH infections include poverty and malnutrition, and STH infections have been associated with delays in cognitive, motor, and social development in children under 5 years of age [3, 4]. The presence of helminths also may cause intestinal blood loss with iron deficiency that manifests as anemia, especially in children with hookworm infection [5].

The World Health Organization and the Pan American Health Organization (WHO/PAHO) set standards for the control of soil-transmitted helminthiasis in Latin America and the Caribbean. These organizations recommend deworming strategies such as preventive chemotherapy (PC), which is an important part of a comprehensive package to eliminate morbidity due to soil-transmitted helminths in populations at risk. This strategy is applied according to the prevalence of soil-transmitted helminths in high-risk areas for preschool-age children (1 to 4 years) and school-age children (5 to 14 years) [6]. In high-risk areas with a prevalence of > 50%, the recommendation is for treatment twice a year (every 6 months), while in low-risk areas with a prevalence > 20 to < 50%, once a year (every 12 months) treatment with albendazole (400 mg) or mebendazole (500 mg) is recommended. In Peru, a universal dose is administered to children between 2 and 11 years of age twice a year in primary care health centers [7].

The efficacy of albendazole is variable against *Trichuris trichiura* compared to other anthelmintics; the cure rate ranges between 2.6 and 64.5% [8] and the egg reduction rate ranges between 7 and 83.1%. However, high cure rates of *Trichuris trichiura* are observed in some contexts [9–11]. In a study conducted in the Peruvian Amazon, an open-pair randomized trial was conducted in fifth-grade children from 18 primary schools (9 intervention and 9 control). Intensity of *Ascaris lumbricoides* infection at follow-up was 58% lower in children from intervention schools, a significant reduction as compared to children from control schools (RR = 0.42; 95% CI 0.21 to 0.85), but no significant changes were observed in the intensity of hookworm or *Trichuris trichiura* [12]. In another study from Peru, a total of 1193 school-age children were dewormed with a single dose of Albendazole (400 mg). Of the 909 children who tested positive for at least one STH infection, a random sample of 385 were followed up 2 weeks later with a second stool sample. The efficacy of albendazole was variable, with an egg reduction rate of 99.8% for *Ascaris lumbricoides* (95% CI 99.3–100); 93.6% for hookworm (95% CI 88.2–96.6), and 72.7% for *Trichuris trichiura* (95% CI 58.5–79, 1) [13]. In many cases treatment with albendazole alone appeared inadequate, especially for *Trichuris trichiura*. Other interventions such as combined therapy have been evaluated in other contexts. Ivermectin results in higher cure rates than albendazole and is well tolerated. The benefits of ivermectin for helminth infections would depend on the amount of resistance present [14–16].

Large-scale interventions to control soil-transmitted helminth infections at the community level with anthelmintic drugs such as albendazole have the potential to exert selective pressures which may favor the development of drug resistance, which could significantly reduce the benefits provided by deworming programs. Drug resistance in parasitic nematodes is caused by a single nucleotide polymorphism (SNP) in the β-tubulin gene at codon positions 200 (T → A), 167 (T → A) or 198 (A → C) [17].

Herein we describe the protocol for our observational study of a prospectively enrolled cohort to assess clinical resistance to albendazole and reinfection by intestinal helminths in children from the Peruvian Amazon receiving treatment according to Peruvian Ministry of Health treatment guidelines [7], to measure preventive treatment effectiveness for 6 months.

### Study design (see Fig. 1)

**Fig. 1:**
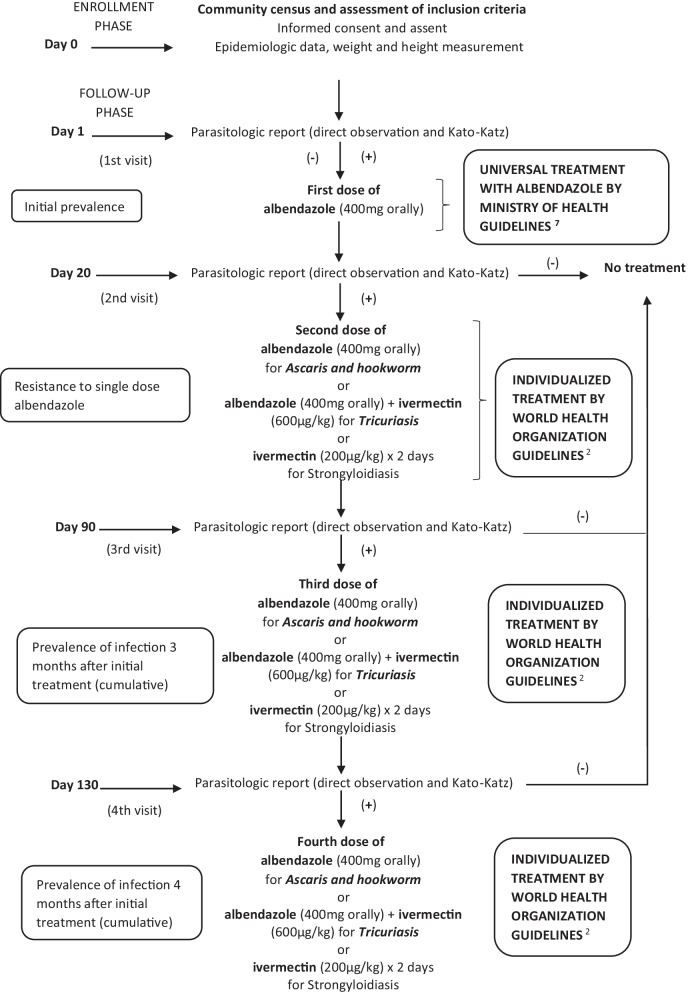
Study design

Prospective cohort study of children aged 2 to 11 years, with an individual time of participation of 4 and a half months.

Participants will be boys and girls ages 2 to 11 years who live in the catchment area of the Centro de Salud 6 de Octubre, located in the district of Belén, city of Iquitos, and department of Loreto in Peru.

Inclusion criteria:

- Parental written informed consent, in addition to the verbal and written assent for children ages 6 to 11 years.
- No antiparasitic treatment in the prior 3 months [18].
- Permanent residence in the study community, with no plans to move in the next 6 months.

Major outcomes:

1. Albendazole resistance of intestinal helminths (Trichuriasis, Ascariasis, Hookworm, Strongyloides).
2. Reinfection with intestinal helminths 4 months after treatment with Albendazole.

### Sample size

Sampling will be carried out throughout the jurisdiction of the 6 de Octubre Health Center, which includes rural, peri-urban and urban areas, with a total potential study population of 3379 children ages 2 to 11 years based on health center databases. Based on this study population of 3379 children aged 2 to 11 years, to measure clinical resistance to albendazole a sample of 380 children was needed. This calculation was made in Epi Info Version 7.2 applying a type I error of 5% and assuming 50% prevalence of helminths in children, 25% frequency of *Trichuris trichiura* in children with helminths, 50% resistance to albendazole based on detection of any helminth in a follow-up sample and adjusting for 10% loss to follow-up for study participants.

### Recruitment of participants

To identify eligible children for the study, a community census is being conducted in the catchment area of the Centro de Salud 6 de Octubre, which includes rural, peri-urban and urban areas. Rural areas are defined as streets located on the opposite side of the Itaya river from the city of Iquitos, which is a flood prone area without basic services, electricity and drinking water. Peri-urban areas are located on the Iquitos side of the river and extend into the river, with houses on stilts or raft houses that float on the surface of the river. Many of these houses already have electricity and drinking water, and they are usually located around the health center. Another part of the study community is located in the urban area of Iquitos, with concrete houses that all basic services and access to food markets, etc.

For enrollment in rural areas, a sweep strategy will be applied, meaning that we will look for children who meet the inclusion criteria throughout the community. For the peri-urban area homes without an address (located on rafts) we will also apply the same strategy. In the peri-urban and urban area where there are well-defined maps with streets and addresses, we will recruit candidates with numerical codes from the community census based on a random number generator, starting from the first block and then moving forward clockwise to the next block.

Once the first randomly selected child of any age between 2 and 11 years has been recruited, enrollment will continue in the same way until the established strata are completed: age 2 years = 36 children; 3 years = 36 children; 4 years = 36; 5 years = 32 children; 6 years = 33 children; 7 years = 34 children; 8 years = 35 children; 9 years = 36 children; 10 years = 33 children; 11 years = 34 children. Once all the blocks of the study community have been sampled, if the target number of participants for each stratum were not completed, the process will be repeated from the beginning and thus complete the corresponding sample for each age group.

## Data and specimen collection (see Fig. 1 for overview of study visits and procedures)

Following informed consent, demographic data will be collected, including housing characteristics, general caregiver information, and information on hygiene and childcare. Weight and height measurements will be performed by a member of the on-site investigation team, and a stool collection container will be provided to the caregiver [19]. Fresh feces (10–20 g) will be collected from each participant and the date and time of specimen collection will be recorded upon retrieval. At the end of the day specimens will be transported to the laboratory in a biological sample transport box with ice at the end of the day, for analyses that will be carried out within the next 24 h.

### Albendazole treatment and clinical follow-up

Following enrollment and initial stool specimen collection, albendazole 400 mg will be administered orally in accordance with Peruvian Ministry of Health guidelines (RM No. 479-2017-MINSA) as part of the universal treatment policy in Peru. Stool samples for the study will be collected three times after the first stool sample is taken. This means that the same procedures will be repeated at 15 to 25 days, 90 to 100 days and at 130 to 140 days (in order to measure resistance and reinfection at 11%, 50% and 75% of the 6-month interval recommended for repeating preventive treatment). If parasites are found in samples after treatment with albendazole, individualized treatment will be administered according to World Health Organization standard of care treatment guidelines [2]. For *Ascaris* and hookworm, albendazole 400 mg will be administered as a single oral dose. For *Trichuris trichiura*, ivermectin 600 µg/kg/day will be administered with albendazole 400 mg, both as single oral doses [20, 21]. For *Strongyloides stercolaris*, Ivermectin 200 µg/kg/day for 2 days will be administered [22–24].

### Laboratory analysis

Fecal samples will be analyzed by direct observation of eggs and/or larvae by microscopy and egg counting by the Kato-Katz technique. Direct microscopy is the diagnostic standard in areas with limited resources. The Kato-Katz method is used to estimate the intensity of infection by *Ascaris lumbricoides*, hookworms and *Trichuris trichiura*. Although this method is more sensitive than direct microscopy, it requires more time and labor to detect geohelminth eggs [25, 26]. Once the procedures have been carried out and the results obtained, the remaining stool samples will be aliquoted and stored at − 80 °C.

In the second sample, molecular detection of helminths by polymerase chain reaction (qPCR) will be performed on specimens with helminths detected (i.e., clinical resistance) in order to determine the presence of resistance markers at the genetic level.

- Direct observation: In a properly labeled slide, 0.5 g of feces will be applied with a wooden applicator and 20 µL of saline solution will be added. The first observation will be made from left to right, then the procedure will be repeated with 20 µL of Lugol’s solution added. Both observations will be made with the 10× and 40× objectives in order to qualitatively identify the absence or presence of helminths (*Ascaris lumbricoides*, *Trichuris trichiura*, hookworms, and *Strongyloides stercoralis*).
- Kato-Katz method: Two grams of feces will be placed on a smooth and sterile surface, covered with a piece of nylon and pressed with a flat-edge plastic applicator. The filtered feces will then be placed in the center of a 41.7 mg template on a slide until the hole is filled, after which the template will be removed and the sample will be covered with a cellophane sheet previously imbued in a glycerol-blue solution of methylene, and pressure will be applied until the sample is distributed homogeneously. After a 30-min incubation, eggs counts will be carried out using the 10× objective and a cell counter, the total number of eggs counts per species will be reported in eggs per gram (epg) and will be multiplied by 24 (41.7 mg template) [27], and the results of each parasite found will be recorded and reported quantitatively. This procedure will be performed on the 4 samples per subject collected throughout the study [28].
- STH infection by qPCR: To measure the intensity of the infection, quantitative polymerase chain reaction (qPCR) will be used in such a way that the Kato-Katz variability between users is reduced and the sensitivity of the detection of low-grade parasitic infections is increased [29, 30]. Real-time multiplex qPCR will be performed on positive samples (Day 0) for helminths that were identified by direct observation and Kato-Katz for the simultaneous detection of *Ascaris lumbricoides*, *Trichuris trichiura*, *Necator americanus*, *Ancylostoma duodenale*, and *Strongyloides stercoralis* [31]. If the sample from Day 0 is negative, the sample from Day 15 will be analyzed if it is positive for helminths. Amplification will be performed in a Quant Studio 7 Flex thermocycler (Applied biosystems) in a total volume of 25 µL using GoTaq® qPCR Probe Master Mix (Promega, USA) and 5 µL of fecal DNA. There will be two multiplex qPCR assays (STH1 and STH2); STH1 will be used to detect *Ascaris lumbricoides*, *Trichuris trichiura* and *Strongyloides stercoralis*, while STH2 will be used for the 3 species of hookworms. The cycling conditions for both cases will be the following: 1 cycle at 95 °C for 5 min, 40 cycles at 95 °C for 10 s and 1 cycle at 60 °C for 60 s. The primers will be the same ones used by Azzopardi et al. [32] which can be seen in Table 1.
  **Table 1:** Primers used in multiplex qPCR analysis of stool samples by STH1 and STH2

qPCR	Target	Primer	Sequence (5′–3′)	Size (bp)	Gene detected	Final conc. (nM)
STH1	*Strongyloides stercoralis*	Strongy-F	GAATTCCAAGTAAACGTAAGTCATTAGC	101	18S	100
		Strongy-R	TGCCTCTGGATATTGCTCAGTTC			100
		Strongy-P	FAM-ACACACCGG/ZEN/CCGTCGCTGC-IBFQ			50
STH1	*Trichuris trichiura*	Tri-F	TTGAAACGACTTGCTCATCAACTT	76	18S	100
		Tri-R	CTGATTCTCCGTTAACCGTTGTC			100
		Tri-P	CY5-CGATGGTAC/TAO/GCTACGTGCTTACCATGG-IBRQ			50
STH1	*Ascaris lumbricoides*	Asc-F	GTAATAGCAGTCGGCGGTTTCTT	87	Internal transcribed spacer 1	100
		Asc-R	GCCCAACATGCCACCTATTC			100
		Asc-P	HEX-TTGGCGGAC/ZEN/AATTGCATGCGAT-IBFQ			50
STH2	*Ancylostoma* spp.	Anc-F	CGGGAAGGTTGGGAGTATC	104	Internal transcribed spacer 1	100
		Anc-R	CGAACTTCGCACAGCAATC			100
	*A*. *duodenale*	Aduo-P	HEX-TCGTTAC + T + GGGTGACGG-IBFQ			50
	*A*. *ceylanicum*	Acey-P	FAM-CCGTTC + CTGGGTGGC-IBFQ			50
STH2	*Necator americanus*	Nec-F	CTGTTTGTCGAACGGTACTTGC	101	Internal transcribed spacer 2	100
		Nec-R	ATAACAGCGTGCACATGTTGC			100
		Nec-P	CY5-CTG+TA+CTA+CG+CAT+TGTATAC-IBRQ			50
STH1 Y STH 2	*Phocine herpes virus*	PhHV-F	GGGCGAATCACAGATTGAATC		gB gene	100
		PhHV-R	GCGGTTCCAAACGTACCAA			100
		PhHV-P	Cy5-TTTTTATGTGTCCGCCACCATCTGGATC-BHQ2			50
- DNA extraction from STH: Nucleic acid extraction will be performed using the purification method by column centrifugation and cell disruption by bead beater, which consists of lysing the sample by mechanical disruption. The guidelines of the modified protocol of the QIAmp Fast DNA Stool mini kit (CAT 51604) will be followed, which consists of 4 phases (lysis, filtering, washing and elution), with an approximate time of 60 min for 8 samples. To evaluate the quality of the extraction and the efficiency of the amplification, extrinsic controls (MS2 and PhHV) will be added to each sample during the lysis phase. Subsequently, the DNA obtained will be stored at − 20 °C for the following processes or at − 80 °C for prolonged storage.

### Statistical analysis

Data will be analyzed in Access using Stata, using chi square, Fisher’s exact, and Wilcoxon tests to compare frequencies between groups. To analyze the data from direct observation and quantification by the Kato-Katz method, descriptive statistics will be used, to report the prevalence found in percentages. For the sociodemographic and clinical data of the study population, measures of central tendency and dispersion will be used for the quantitative variables and measures of relative frequency for the qualitative variables. Comparisons will also be made between sample 1, sample 2, sample 3 and sample 4 to determine the positivity of each species of parasite found using the Yates chi-square test, setting a significance level of p < 0.05.

The sociodemographic variables will be compared with the parasite load and presence/absence of parasites, using a multiple logistic regression model (univariate t-test), to determine the variables statistically associated with infection by *Ascaris lumbricoides*, *Trichuris trichiura* and hookworms.

The quantification performed with the Kato-Katz method will be compared with the Ct value (Ct threshold) of the multiplex qPCR, by converting Log10 of the absolute egg count per stool sample, in order to estimate the absolute egg counts in eggs per gram (EPG) in the DNA extractions, using the following formula [33]:

$${\text{EPG}}\;{\text{Ascaris}} = 10\left( {{\text{Ct}} - 36.97} \right)/ - 3.489,$$

$${\text{EPG}}\;{\text{Trichuris}} = 10\left( {{\text{Ct}} - 36.73} \right)/ - 3.288,$$

$${\text{EPG}}\;N.\;americanus = 10\left( {{\text{Ct}} - 35.02} \right)/ - 3.641,$$

$${\text{EPG}}\;A.\;duodenale = 10\left( {{\text{Ct}} - 35.02} \right)/ - 3.641.$$

To determine treatment efficacy, the prevalence will be measured before and after receiving treatment and compared using the Yates chi-square test. For calculations of cure rate and intensity of infection obtained from qPCR, only data from positive results before taking the first dose will be included [34].

The cure rate will be measured using the following formula:

$$\frac{{\# \;{\text{positive}}\;{\text{for}}\;{\text{helminths}}\;\left( {{\text{pretreatment}}} \right) - \# \;{\text{negative}}\;{\text{for}}\;{\text{helminths}}\;\left( {{\text{post}}\;{\text{treatment}}} \right)}}{{\# \;{\text{cases}}\;{\text{positive}}\;{\text{for}}\;{\text{helminths}}\;\left( {{\text{pretreatment}}} \right)}} \times 100.$$

Intensity of infection will be measured using the following formula:

$$\frac{{{\text{mean}}\;{\text{egg}}\;{\text{count}}\;\left( {{\text{pretreatment}}} \right) - {\text{mean}}\;{\text{egg}}\;{\text{count}}\;\left( {{\text{post}}\;{\text{treatment}}} \right)}}{{{\text{mean}}\;{\text{egg}}\;{\text{count}}\;\left( {{\text{pretreatment}}} \right)}} \times 100.$$

And the calculation of the rate of reduction of eggs by helminth species:

$$\frac{{\left[ {1 - {\text{mean}}\;{\text{egg}}\;{\text{count}}\;{\text{in}}\;{\text{EPG}}\;\left( {{\text{post}}\;{\text{treatment}}} \right)} \right]}}{{{\text{mean}}\;{\text{egg}}\;{\text{count}}\;{\text{in}}\;{\text{EPG}}\;\left( {{\text{pretreatment}}} \right)}} \times 100.$$

In addition, a comparison of the cycle threshold (Ct) by qPCR will be made using the Student’s t-test for paired samples.

## Discussion

Data from observational clinical efficacy studies such as this one are vitally important to assure continued high levels of geohelminth infection control, and to permit re-evaluation of clinical guidelines when necessary to achieve desired program goals.

## Data Availability

Data generated from this study will be available from the corresponding author on reasonable request, or published with the final results, after all findings are available. Data will be shared after approval of a proposal by the authors for legitimate scientific purposes.

## Abbreviations

STH: Soil-transmitted helminths
WHO: World Health Organization
PAHO: Pan American Health Organization
PC: Preventive chemotherapy
qPCR: Quantitative polymerase chain reaction
STH1 and STH2: Two multiplex qPCR assays for detection of geohelminths
DNA: Deoxyribonucleic acid
